# Identification of Multiple Low-Level Resistance Determinants and Coselection of Motility Impairment upon Sub-MIC Ceftriaxone Exposure in Escherichia coli

**DOI:** 10.1128/mSphere.00778-21

**Published:** 2021-11-17

**Authors:** Carly Ching, Muhammad H. Zaman

**Affiliations:** a Boston University, Department of Biomedical Engineering, Boston, Massachusetts, USA; Escola Paulista de Medicina/Universidade Federal de São Paulo

**Keywords:** *E. coli*, antibiotic resistance, ceftriaxone

## Abstract

Resistance to third-generation cephalosporins among Gram-negative bacteria is a rapidly growing public health threat. Among the most commonly used third-generation cephalosporins is ceftriaxone. Bacterial exposure to sublethal or sub-MIC antibiotic concentrations occurs widely, from environmental residues to intermittently at the site of infection. Quality of ceftriaxone is also a concern, especially in low- and middle-income countries, with medicines having inappropriate active pharmaceutical ingredient (API) content or concentration. While focus has been largely on extended-spectrum β-lactamases and high-level resistance, there are limited data on specific chromosomal mutations and other pathways that contribute to ceftriaxone resistance under these conditions. In this work, Escherichia coli cells were exposed to a broad range of sub-MICs of ceftriaxone and mutants were analyzed using whole-genome sequencing. Low-level ceftriaxone resistance emerged after as low as 10% MIC exposure, with the frequency of resistance development increasing with concentration. Genomic analyses of mutants revealed multiple genetic bases. Mutations were enriched in genes associated with porins (*envZ*, *ompF*, *ompC*, and *ompR*), efflux regulation (*marR*), and the outer membrane and metabolism (*galU* and *pgm*), but none were associated with the *ampC* β-lactamase. We also observed selection of *mgrB* mutations. Notably, pleiotropic effects on motility and cell surface were selected for in multiple independent genes, which may have important consequences. Swift low-level resistance development after exposure to low ceftriaxone concentrations may result in reservoirs of bacteria with relevant mutations for survival and increased resistance. Thus, initiatives for broader surveillance of low-level antibiotic resistance and genomic resistance determinants should be pursued when resources are available.

**IMPORTANCE** Ceftriaxone is a widely consumed antibiotic used to treat bacterial infections. Bacteria, however, are increasingly becoming resistant to ceftriaxone. Most work has focused on known mechanisms associated with high-level ceftriaxone resistance. However, bacteria are extensively exposed to low antibiotic concentrations, and there are limited data on the evolution of ceftriaxone resistance under these conditions. In this work, we observed that bacteria quickly developed low-level resistance due to both novel and previously described mutations in multiple different genes upon exposure to low ceftriaxone concentrations. Additionally, exposure also led to changes in motility and the cell surface, which can impact other processes associated with resistance and infection. Notably, low-level-resistant bacteria would be missed in the clinic, which uses set breakpoints. While they may require increased resources, this work supports continued initiatives for broader surveillance of low-level antibiotic resistance or their resistance determinants, which can serve as predictors of higher risk for clinical resistance.

## INTRODUCTION

Antibiotic resistance to third-generation cephalosporins among Gram-negative bacteria is a growing public health challenge (1, 2). Ceftriaxone is one of the most commonly dispensed antibiotics globally (3–5). Broadly, β-lactam antibiotics bind to penicillin binding proteins (PBPs), which mediate cross-linking of peptidoglycan, a major component of the cell wall (6). While focus has largely been on clinical resistance and the role of plasmid-borne extended-spectrum β-lactamases (ESBLs), enzymes which can hydrolyze third-generation cephalosporins (7), or hyperproduction of the chromosomal AmpC β-lactamase (8, 9), there are limited data on nonenzymatic chromosomal mutations associated with ceftriaxone resistance. However, one study found that of clinical cephalosporin-resistant bacterial isolates from a nationwide clinical collection in Norway, a high proportion (7/35 Escherichia coli isolates and 8/11 Klebsiella pneumoniae isolates) had an indecisive mechanism of resistance (not related to β-lactamases or porins) (10). Overall incidences of cephalosporin resistance with an indecisive mechanism out of the total collection tested were 0.4% (*n* = 2213) and 3% (*n* = 303) for E. coli and K. pneumoniae, respectively (10). Determining mutations that help bacteria survive and contribute to ceftriaxone resistance is important to better identify and understand resistance emergence.

Bacterial exposure to sublethal concentrations or sub-MICs of antibiotics occurs in numerous situations, from residues in the environment and wastewater effluent to intermittently at the site of infection in the body (11) and upon use of poor-quality antibiotics (12). Ceftriaxone, which is typically taken intravenously as a solution, has ongoing quality issues with low active pharmaceutical ingredient (API) content (12, 13). Values as low as 55% of the stated API content have been detected in injection ceftriaxone sodium, with various percent concentrations in between 55% and 100% reported (14, 15). Furthermore, ceftriaxone solutions are very unstable under improper storage conditions (16). Importantly, since the body may reach various sub-MIC levels across treatment, this will further decrease in tandem with poor-quality or degraded drugs. We previously found that low-level multidrug resistance developed upon exposure to a broad range of sub-MIC ciprofloxacin concentrations in E. coli, in a concentration-dependent manner. This broad resistance was afforded by single mutations in efflux regulators (17). Notably, these low-level resistances are more difficult to detect and would be missed with clinical breakpoints. This has important implications, namely, the development of a reservoir of bacteria prone to survival and higher-level resistances that is not reported during surveillance or in antibiograms. We hypothesized that this may be a wider issue and that sub-MIC exposure to other antibiotic classes, in this case the β-lactam ceftriaxone, may result in a similar magnitude change in resistance through nontarget mutations.

In this study, we investigated the impact of exposure to a broad range of sub-MIC ceftriaxone concentrations against E. coli, a common agent of ceftriaxone-resistant infections, especially urinary tract infections (18). While some evidence suggests that sublethal concentrations of β-lactam antibiotics have a role in resistance and mutagenesis (19–23), there are limited data on the role of sublethal selective windows of ceftriaxone on resistance and on which resistant determinants are most relevant under these concentrations. It is important to fill this gap, in order to better identify resistance emergence in the environment and clinic, where low levels of antibiotics are pervasive. This work can help identify resistance patterns, bacterial physiology and causative mechanisms, and novel resistant markers upon exposure to sublethal ceftriaxone concentrations. This can inform holistic recommendations to monitor and prevent resistance due to low levels of antibiotics in the environment and clinic.

## RESULTS

### Growth decreases with increasing sub-MIC and emergence of mucoid phenotype at higher concentrations.

**FIG 1:**
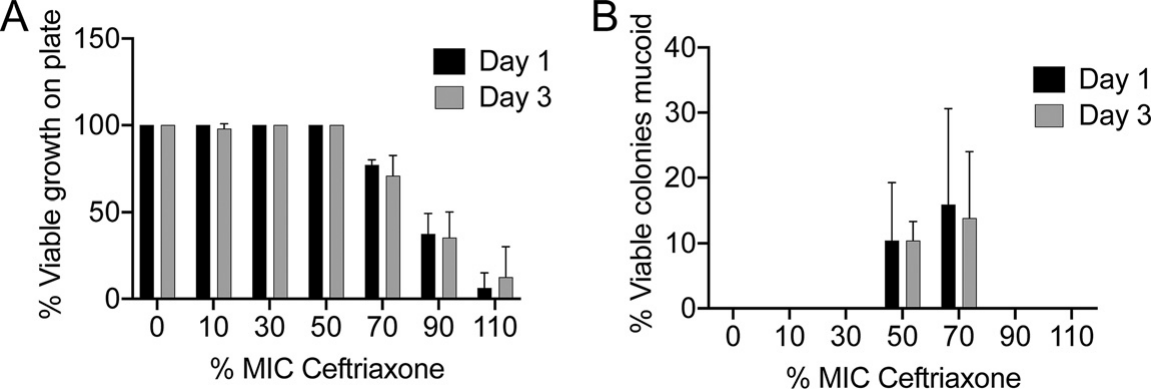
(A) Mean percentage of individual wells that displayed viable growth plotted against exposure concentration of ceftriaxone (as percent MIC) after day 1 and day 3 of passaging. Error bars represent the standard deviations from 2 independent experiments (*n* = 48 in total). (B) Mean percentage of wells with viable growth that displayed a mucoid phenotype on LB agar incubated at room temperature (RT) for 96 h after day 1 and day 3 of passaging. Error bars represent the standard deviations from 2 independent experiments (*n* = 48 total for 50% MIC and *n* = 37 for 70% MIC on day 1 and *n* = 34 for 70% MIC on day 3).

To begin, E. coli MG1655 cells were exposed to concentrations corresponding to 10 to 110% of the MIC (0.0625 μg/ml), in 20% intervals, along with a drug-free control condition. This gradient of concentrations displayed consistent decreases in survival (Fig. S1A). During this experiment, we observed the appearance of hypermucoid colonies after incubation at room temperature (RT). To determine the frequency of this mucoid phenotype, we performed experiments using 24-well plates to test 24 samples at each concentration per independent experiment. Bacteria were passaged in fresh medium containing the corresponding ceftriaxone concentration after 24 h of exposure, for 3 consecutive days. The frequency of wells displaying viable growth decreased with increasing exposure concentration, as expected (Fig. 1A). At day 1 and day 3, samples were patched on drug-free media and incubated at RT. We observed the appearance of mucoid colonies at 50% and 70% MIC exposure (Fig. 1B).

10.1128/mSphere.00778-21.1 FIG S1 (A) Percent survival (relative to that with no drug treatment) plotted against ceftriaxone exposure concentration (as percent MIC) from viable cell measurements after 24 h of exposure. Error bars represent standard deviations from duplicate measurements. (B) Mean fold increase in MIC (relative to WT) for each individual isolate tested. Ceftriaxone exposure concentration (percent MIC) is indicated at the bottom. Error bars represent standard deviations from duplicate technical measurements. (C) (i) Diameter relative to that of the WT for each isolate tested. Ceftriaxone exposure concentration (percent MIC) is indicated at the bottom. (ii) Assay was performed independently twice to test consistency due to subtle differences in soft agar made on different days. Relative diameter measurements of each independent assay (1 or 2) matched. Download FIG S1, TIF file, 1.0 MB. Copyright © 2021 Ching and Zaman. 2021 Ching and Zaman. https://creativecommons.org/licenses/by/4.0/ This content is distributed under the terms of the Creative Commons Attribution 4.0 International license.

### Low-level stable resistance (increase in MIC) emerges after 30% MIC exposure.

**FIG 2:**
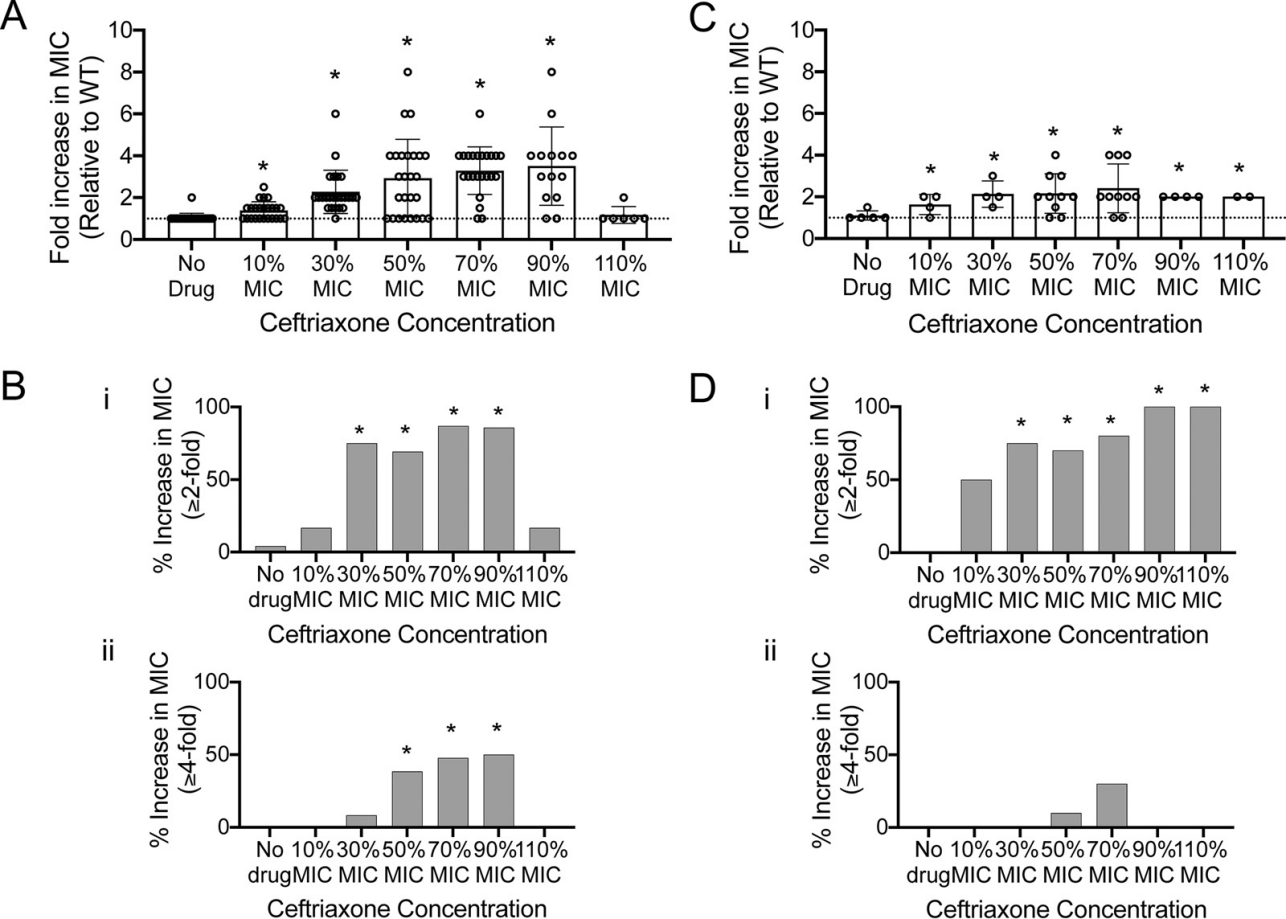
(A) Mean fold increase (relative to parental WT cells) in MIC of ceftriaxone plotted against ceftriaxone concentration (as percent MIC) after 3-day exposure. (B) Percentage of isolates tested with ≥2-fold (i) or ≥4-fold (ii) and over increase in MIC. (C) Mean fold increase (relative to WT cells) in MIC of ceftriaxone of passaged isolates against ceftriaxone concentration. For panels A to C, error bars represent standard deviations from 2 independent experiments. Each dot represents the mean of one replicate, measured in technical duplicate. A fold change of 1 indicates no change. (D) Percentage of passaged isolates with ≥2-fold (i) or ≥4-fold (ii) and over increase in MIC. For panels A and B, *n* = 24 for no drug to 30% MIC conditions, *n* = 26 for 50% MIC, *n* = 23 for 70% MIC, *n* = 14 for 90% MIC, and *n* = 6 for 110% MIC. For panels C and D, *n* = 5 for no drug, *n* = 4 for 10%, 30%, and 90% MIC, *n* = 10 for 50% and 70% MIC, and *n* = 2 for 110% MIC. For panels A and C, an unpaired two-tailed *t* test was used for statistical analysis of mean fold change compared to the no-drug condition (*, *P* < 0.05). For panels B and D, statistical differences between no-drug and exposure groups were determined by two-tailed Fisher’s exact text (*, *P* < 0.05).

We next measured antibiotic susceptibility of 12 isolates per exposure concentration from each independent experiment after 3-day exposure to ceftriaxone and growth in drug-free media overnight. Change in ceftriaxone MIC emerged after 10% MIC exposure, with the frequency of resistant isolates increasing with drug concentration, with a significant difference in average fold increase in MIC (relative to that for untreated wild-type [WT] cells) occurring between the no-drug and 10% MIC exposure groups and between the 10% MIC and 30% MIC exposure groups (*t* test, *P* value < 0.05 [Fig. 2A and B]). Thus, even though they are low (∼2- and 4-fold), we see reproducible increases in MIC after ceftriaxone exposure that are not explained by the variance of assay provided by the no-drug control (*P* value < 0.05 [Fig. 2A and C]). After 3 days, cells exposed to 30% MIC and 50% MIC ceftriaxone were more likely to show ≥2-fold and ≥4-fold increases in MIC, respectively, than those under the no-drug condition (control) (Fisher’s exact text, *P* value < 0.05 [Fig. 2B]). To test whether the MIC changes were stable, we passaged two or more of the most resistant isolates from each exposure concentration, per experiment, on drug-free media for 5 days, in addition to all mucoid isolates (Table S1; see MIC and mucoid status before passage). For conditions where there were fewer than two isolates that had a change in MIC, isolates displaying no change were passaged. In total, 39 isolates were passaged (Table S1). After passage, cells originally exposed to 30% MIC and above had a significant increase (*t* test, *P* value < 0.05) in MIC compared to the no-drug control, albeit low level (Fig. 2C), with the frequency of isolates with a ≥2-fold increase in MIC increasing significantly after 30% MIC exposure compared to the no-drug control (Fig. 2D, panel i). The majority of MIC increases were below 4-fold (Fig. 2D, panel i versus panel ii). The ceftriaxone MIC for each individual passaged isolate is provided in Fig. S1B.

10.1128/mSphere.00778-21.2 TABLE S1 Complete sequencing and phenotypic details of sequenced isolates. Download Table S1, XLSX file, 0.02 MB. Copyright © 2021 Ching and Zaman. 2021 Ching and Zaman. https://creativecommons.org/licenses/by/4.0/ This content is distributed under the terms of the Creative Commons Attribution 4.0 International license.

### Changes in motility enriched with low-level resistance.

**FIG 3:**
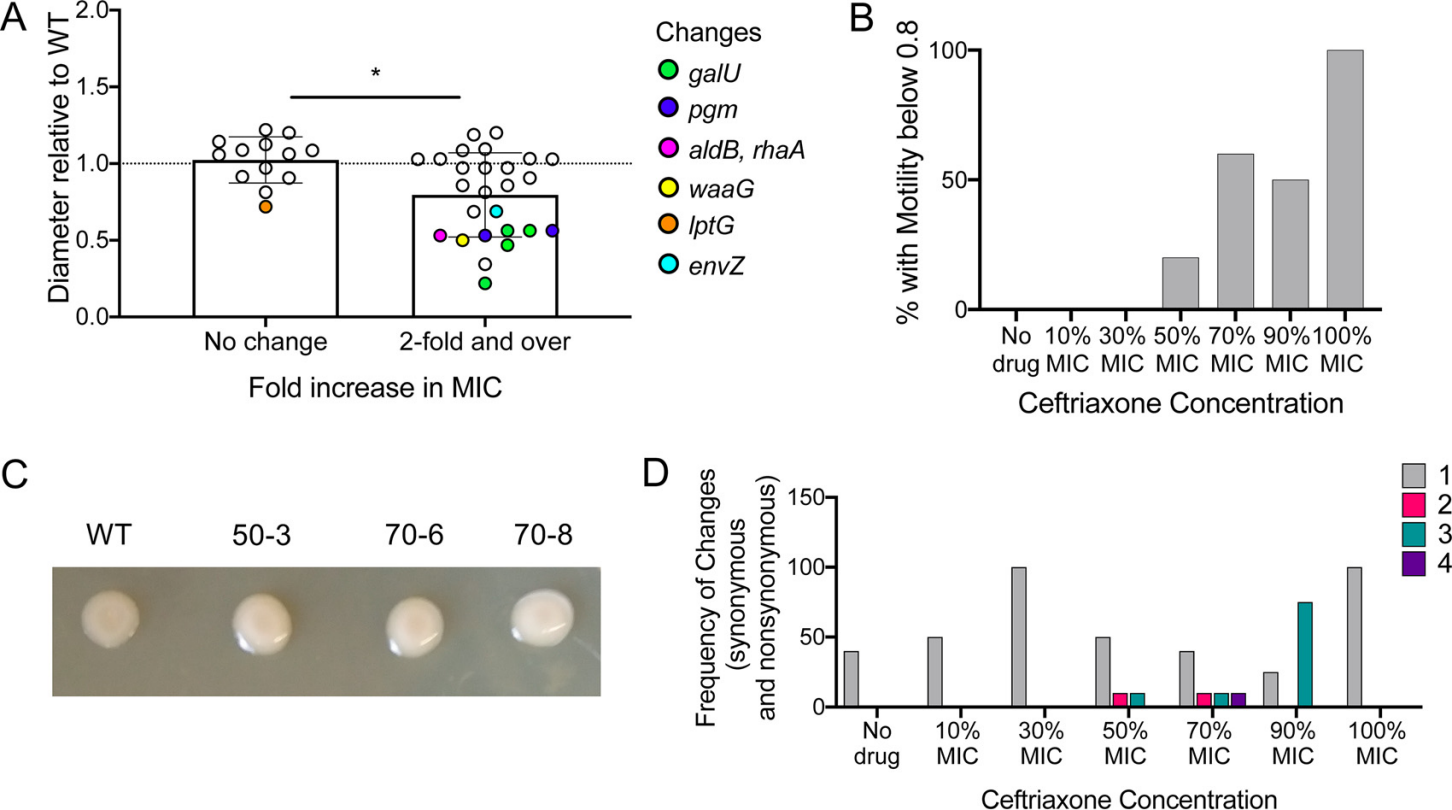
Diameter relative to that of the WT is plotted against change in resistance. Genetic changes of mutants with relative diameter below 0.8 are highlighted. Resistance is defined as fold increase greater than or equal to 2. An unpaired two-tailed *t* test was used for statistical analysis to compare mean motility ratio between resistant and susceptible isolates. *, *P* < 0.05. (B) Percent with motility impairment (relative diameter below 0.8) plotted against ceftriaxone concentration. (C) Mucoid isolates (with mutant number) on LB agar compared to the WT after incubation at RT for 96 h. (D) Frequency of number of synonymous or nonsynonymous changes plotted against exposure concentration. For panels A, B, and D, *n* = 5 for no drug, *n* = 4 for 10%, 30%, and 90% MIC, *n* = 10 for 50% and 70% MIC, and *n* = 2 for 110% MIC.

Temperature-dependent mucoid phenotypes are often associated with the RcsCDB phosphorelay system, which also regulates motility (24). We thus tested swimming motility on soft agar of each passaged isolate (Fig. S1C). Grouping motility of isolates against their change in MIC, we observed a significant correlation in impaired motility and ceftriaxone resistance (defined as fold increase in MIC of ≥2) (Fig. 3A). In general, at higher concentrations, more decreases in motility were also observed (Fig. 3B). After passage of 10 mucoid isolates observed initially after antibiotic exposure, 3 maintained a mucoid phenotype (Fig. 3C). Thus, changes in motility were more prevalent and not directly coupled with a mucoid phenotype. However, all mucoid isolates also had impaired motility.

### Multiple genetic changes identified.

To identify the genetic mechanisms underlying resistance and motility phenotypes of all passaged isolates described above (3-day exposure to ceftriaxone, followed by 5-day passage on drug-free media), we performed whole-genome sequencing (WGS). Within this set of 39 isolates, 5 from the control condition (3 days with no drug) were sequenced. The results of enriched genes with changes after selection are summarized in Table 1. We detected three nucleotide changes in our WT parental strain compared to the reference genome, listed in Table S1, along with full data. All isolates sequenced also had these same changes. The frequency of mutations (single nucleotide variant [SNV], insertion, or deletion) increased with concentration (Fig. 3D). Of the isolates from the no-drug condition, 1/5 had a single missense SNV, not associated with genes enriched for mutations under ceftriaxone pressure. Analysis of amino acid substitutions with PROVEAN predicted that all missense SNV mutations that occurred in enriched genes were deleterious, whereas 50% of the missense SNV mutations in nonenriched genes were neutral. No mutations occurred within or in the promoter region of *ampC* (Table S1). The genes that had multiple independent mutations can be grouped into the categories below.

**TABLE 1:** Mutated genes (with frequency of >2) and their biological roles

Gene	GO^*a*^ no., biological process(es)	Mutants (*n* = 39)	Specific mutations (no. of times occurring, if >1)^*b*^	Range of observed mean MIC fold increases among mutants (avg MIC)	Impaired motility/mucoid^*c*^
*ompF*	GO:0006811, ion transport	10-4, 30-1, 30-2, 50-7	W236*, E306*, D29fs, 120 nt upstream^+^	1.5–2 (1.75)	−/−
*ompC*	GO:0006811, ion transport	70-6, 70-9	Q143*^+^, s^+^	4 (4)	−/−
*ompR*	GO:0000160, phosphorelay signal transduction system; GO:0006355, regulation of transcription, DNA templated	50-2, 70-9	M197R, R150C^+^	2–4 (3)	−/−
*envZ*	GO:0000160, phosphorelay signal transduction system	50-1, 70-9	E212V, S242G^+^	3–4 (3.5)	+ (1/2)/−
*baeR*	GO:0000160, phosphorelay signal transduction system; GO:0006355, regulation of transcription, DNA templated	90-3, 90-4	L25W^+(1)^(3), G103A^+^	2 (2)	−/−
*marR*	GO:0006355, regulation of transcription, DNA templated; GO:0046677, response to antibiotic	50-6, 50-10, 70-6, 70-9	T72fs, Q23fs, V66A^+^, Q23fs^+^	1.5–4 (3.375)	−/−
*galU*	GO:006011, UDP-glucose metabolic process; GO:0009058, biosynthetic process	70-4, 90-1, 110-1, 110-2	L120*^+(2)^(3), Q274*	2–4 (2.5)	+ (4/4)/−
*pgm*	GO:0005975, carbohydrate metabolic process; GO:006006, glucose metabolic process	70-5, 90-2	P121L, F266L	2 (2)	+ (2/2)/−
*mgrB*	GO:0070298, negative regulation of phosphorelay signal transduction system	30-3, 90-3	C28F, V11fs^+^	1.5–2 (1.75)	−/−

a GO, gene ontology.

b *, stop codon; s, synonymous; fs, frameshift. Superscript “+” indicates that the mutation is in combination with other nonsynonymous mutations, and number in parentheses indicates no. of times occurring.

c “–” or “+” (frequency of mutants showing phenotype) is used to represent if a phenotype is absent or present, respectively, in any of the mutants. Full data are in Fig. S1 and Table S1.

### Porin genes.

Numerous mutations were observed in porins (*ompC* [2 mutations] and *ompF* [4 mutations]) (25) and regulators of porins (*envZ* [2 mutations] and *ompR* [2 mutations]) (26) (Table 1). In Gram-negative cells, most β-lactams cross the outer membrane by diffusion through the porin proteins OmpC and OmpF. Single point mutations can reduce the channel size or alter electrostatic interactions in the channel, decreasing permeability and reducing antibiotic susceptibility (27). The majority of mutations in OmpC and OmpF were nonsense (Table S1 and Table 1). Notably, data suggest that loss of porin activity is an important clinical mechanism of resistance (28). We observed that this mechanism is selected for at very low API concentrations.

EnvZ-OmpR is a two-component system that positively regulates OmpC and OmpF in response to osmolarity changes. Phosphorylation of the activator OmpR by EnvZ stimulates transcription of *ompC* and *ompF* (26). The selected mutations in *envZ* (E212V and S242G) are just outside and within the catalytic domain (residues 223 to 289) (29) (Table 1). The mutations in *ompR* (M197R and R150C) are in the C-terminal domain. Specifically, M197 is within residues 190 to 200, which comprise the α loop in the helix-turn-helix (HTH) DNA-binding motif (30). A mutation at R150 (R150S) has been shown to disrupt DNA binding through a predicted structural perturbation (30).

### Motility mutants with changes in outer membrane and metabolism.

Mutants with impaired motility were enriched with mutations in *galU* (4 mutations, all nonsense) and *pgm* (2 mutations) (Fig. 3A and Table 1). *galU* (UTP–glucose-1-phosphate uridylyltransferase) catalyzes the synthesis of UDP-glucose, which is a central precursor for lipopolysaccharides (LPS) and osmoregulated periplasmic glucans (OPG) (31). E. coli *galU* mutants have an outer membrane with an incomplete LPS layer and decreased flagellin (32). *pgm* (phosphoglucomutase) is involved in sugar metabolism and the first step of UDP-glucose biosynthesis (33). *pgm* mutants have demonstrated a defect in swimming motility (34). Flagellum formation involves interaction with the outer membrane; thus, mutations that alter the outer membrane and have a role in metabolism and energy production serve to impair motility (35). In addition to the impact on motility, alterations in LPS composition are hypothesized to affect outer membrane barrier properties and antibiotic permeation (36).

Additionally, six other isolates had decreased motility (Fig. 3A). One had missense mutations in *aldB* (aldehyde dehydrogenase B) and *rhaA* (l-rhamnose isomerase), both involved in metabolism, although the *aldB* mutation is predicted to be neutral. This strain was also mucoid (Fig. 3A and C, 50-3). Another motility mutant, which was also mucoid (Fig. 3A and C, 70-6), had a frameshift mutation in *waaG* (lipopolysaccharide glucosyltransferase I), again involved in LPS biosynthesis (37). E. coli *waaG* mutants have been shown to have disabled flagellum biosynthesis (38), to produce colanic acid (24), and to have truncated LPS and a destabilized outer membrane (39). Another mutant with a mild motility impairment had a mutation in *lptG*, which encodes an inner membrane component of the LPS transport system. Decreased LptG increases outer membrane permeability (40). Another mutant had a mutation in *envZ*. Notably, two mutants without any changes displayed impaired motility (and one was also mucoid). These may be due to changes in the expression level.

### Other genes with multiple independent selection events.

Four mutants had changes in *marR*. MarR is a transcription repressor of the *marA*, which positively regulates the AcrAB-TolC efflux pump (41, 42). Three mutations were frameshift mutations, and one was at residue 66 (Table 1). Amino acids 61 to 121 in *marR* are required for its DNA binding activity (43).

BaeR belongs to the BaeSR two-component system, which is also in the OmpR family. Phosphorylated BaeR activates transcription of the *mdtABC* and the *acrD* multidrug efflux pump genes (44, 45). Recent work also found that BaeR overexpression can also regulate expression of outer membrane proteins (46). The mutations in *baeR* (L25W and G103A) (Table 1) are both in the N-terminal receiver domain (47). The mutation L22Q in *baeR* has been previously shown to increase *acrD* and *mdtA* expression in E. coli (48).

Two changes in *mgrB* were selected for independently, after exposure to 30% and 90% MIC ceftriaxone. MgrB is a membrane peptide that functions as a negative-feedback regulator of the PhoP/PhoQ system (49); however, the biological functions of MgrB are largely unknown (50). New data suggest that MgrB affects acid resistance but not completely through PhoP/PhoQ (50). Interestingly, PhoP/Q is regulated by the small RNA MicA in addition to MgrB (51), and MicA also regulates OmpA protein expression (52).

## DISCUSSION

Here, we report multiple genetic changes that develop and are selected for under sub-MIC ceftriaxone exposure in E. coli. There are currently limited full genomic analyses of mutants obtained after ceftriaxone exposure; thus, unlike for other antibiotics, point mutations are not well documented. No mutations for the *ampC* β-lactamase (gene or promoter region) were selected for under sub-MIC conditions. Instead, porins served as a major mechanism for low-level resistance at these concentrations. This finding matches observations from the clinic where 44% of 45 β-lactam (including cefotaxime)-resistant clinical Enterobacter aerogenes isolates had deficient porin activity, and not all had β-lactamase activity (28). This work also highlights the role for nonenzymatic mechanisms related to the outer membrane. While deletion of *galU* has been observed as an important first event in evolution of resistance to ceftazidime or ceftazidime-avibactam in Pseudomonas aeruginosa (53), mutations in other genes (*pgm* and *waaG*) have not been described in the literature as cephalosporin resistance determinants, to our knowledge. We also observed enriched selection of mutations in *mgrB* after subinhibitory ceftriaxone exposure. Mutations in *mgrB* have not been implicated in β-lactam selection or resistance development in E. coli, to our knowledge. Instead, *mgrB* has been identified as a determinant of colistin resistance in other pathogens, but not in E. coli (54).

Many of the changes that were enriched for after selection with ceftriaxone can contribute broadly to resistance. Porins also mediate transport of other antibiotics across the outer membrane, increasing the risk of multidrug resistance (55). Indeed, OmpF mutants are resistant to multiple antibiotics (56), and a *galU* deletion and point mutant had a 2-fold increase in MIC to fosfomycin (57). One study found that 42.9% of clinically ceftriaxone-resistant uropathogenic E. coli (UPEC) isolates were multidrug resistant. This study checked only for targeted resistance genes and did not include those found in this study (58). With increased accessibility to genomic sequencing, such that in certain countries WGS is less expensive than conventional microbiology (59), expanded genomic surveillance to identify other resistance markers or accompanying mutations could be important to identify resistance patterns or reservoirs.

Numerous isolates had multiple mutations, with gene combinations selected for independently. In the absence of β-lactamases, high-level carbapenem resistance has been shown to occur from a combination of mutations in *envZ*, *ftsI* (PBP3), *mrdA* (PBP2), *acrB*, and *acrR* after carbapenem exposure (60). Additionally, there may be cross talk between two-component systems (46). The varied genetic changes support evidence that antibiotic resistance evolution can occur via several parallel pathways (60). Increasing antibiotic sub-MICs suggest increasing selective pressure, as resistance frequency and other phenotypic changes are positively correlated (Fig. 2 and 3). At higher concentrations, an increased number of mutations are selected for (Fig. 3D). Notably, alterations in porins were selected for at 10% to 90% MIC, showing that this is a major resistance mechanism, even under very low levels of the antibiotic.

We observed coselection of resistant mutants that had impaired motility (Fig. 3A). This is generally explained by alterations in genes involved with the outer membrane, which changes to can affect motility (35). However, a previous transposon mutagenesis screen found that disruptions beneficial to growth in ampicillin and cefoxitin were in genes specific to flagellum assembly (i.e., flagellar biosynthesis and motor switch proteins), which supports a benefit specifically for motility itself (61). We also observed transient and stable mucoid phenotypes in cells exposed to low levels of ceftriaxone (Fig. 3C). The former may be due to induction of the *rcs* pathway, which is upregulated after β-lactam exposure (62). These phenotypic changes may impact other processes such as virulence or horizonal gene transfer. Mucoid cells are associated with virulence (63), and OmpR regulates virulence in addition to outer membrane proteins (64); thus, besides resistance, there may be selection for host interaction effects. The link between motility and horizontal gene transfer, specifically plasmid conjugation, is less defined; however, data suggest that motility is reduced when conjugation of certain plasmids is prompted (65). Because plasmid ESBLs are a major concern, conferring a phenotype that can impact conjugation will have serious consequences. In future work it will be important to establish the possible clinical impact of these mutations by using animal models of infection.

Low-level resistance (increases in MIC) emerged after just 3 days (Fig. 2), at as low as 10% MIC, due to stable genetic changes. Most often, surveillance at both the global and hospital levels is for clinical antibiotic resistance, which ignores levels of resistance such as those observed in this study, which are below the clinical breakpoints based on epidemiologic cutoff values. However, these bacteria may indeed have relevant mutations which promote survival under antibiotic treatment and lead to reservoirs for resistant bacterial outbreaks (66, 67). Our findings show that this broadly continues to be an oversight in the effort to tackle the global challenge of antimicrobial resistance. Notably, breakpoints are continuously being lowered (68), suggesting that monitoring smaller changes is important.

To be proactive about antibiotic resistance development and outbreaks of resistant infections, it is important to begin incorporating low-level resistance into surveillance for multiple classes of antibiotics. This is especially relevant in regions that have a high burden of antibiotic resistance or contributing factors that result in low antibiotic exposure and subsequently resistant mutations (indiscriminate antibiotic usage, high agricultural burden, or high prevalence of poor-quality antibiotics), not just in clinical settings but in the environment and animals as well. The existing framework for qualitative and quantitative surveillance of low-level resistance can be reviewed (8) and expanded. While resources and time for conducting quantitative susceptibility studies for low-level resistances may be limited, genomic sequencing for nontarget resistance determinants may also serve as an important tool for surveillance (59). Furthermore, stewardship and initiatives to reduce these contributing factors need continued effort.

## MATERIALS AND METHODS

### Strains and culture conditions.

E. coli MG1655 (ATCC 700926) was used for all experiments. All cultures were routinely grown in LB medium and incubated at 37°C with shaking at 180 rpm for liquid cultures, unless otherwise noted. Ceftriaxone (Acros Organics; lot A0394235) solutions were made in sterile deionized water and added to the medium as indicated. Fresh solutions were made for daily use. All strains used are described in Table S1.

### Ceftriaxone treatment and susceptibility and survival measurements.

The MIC was determined to be 0.0625 μg/ml through broth microdilution performed using CLSI guidelines (69). For ceftriaxone passaging experiments, saturated liquid cultures were diluted in LB broth and grown to exponential phase until turbidity matched a 0.5 McFarland standard (∼5 × 10^8^ CFU/ml). Each well of a 24-well polystyrene plate (noncell culture treated) was inoculated with 1 ml of cells at a 1:1,000 dilution into LB broth containing no drug or 10 to 110% (in 20% increments) of the ceftriaxone MIC for 24 h. Each plate represented the same drug concentration (*n* = 24 for each concentration). After 24 h, 2 μl of cells from each exposure condition was added to 1 ml of fresh medium containing the corresponding initial treatment (i.e., cells treated with 10% MIC were subcultured into fresh medium containing 10% MIC) for another 24 h. This passaging was repeated once more. At day 1 and day 3, cells were patched onto drug-free LB agar and incubated at room temperature (RT) for up to 96 h. These plates were checked for visibly mucoid colony phenotypes. Experiments were performed independently, in biological duplicate. To determine the MIC of cells after antibiotic exposure, cells were outgrown in drug-free media overnight and subsequently used in a standard microplate broth dilution MIC (range, 2 to 0.0312 μg/ml, plus a no-drug control) using saturated cells (70). The MIC of stock WT cells was determined concurrently for all MIC assays. For each independent experiment, measurements were performed in technical duplicate. All fold changes in MIC were expressed relative to that for stock parental WT cells (no passaging). An unpaired two-tailed *t* test was used for statistical analysis to compare mean fold change in MIC between exposure groups (a *P* value of <0.05 was considered statistically significant). Survival was measured as before (17). Two-tailed Fisher’s exact test was used to compare the variables (proportion above or below fold increase cutoff) between no-drug control group and experimental group (a *P* value of <0.05 was considered statistically significant).

### Drug-free passaging and MIC measurements.

At least two isolates that displayed the highest change in ceftriaxone MIC from each exposure concentration after 3 days per experiment (i.e., at least four isolates per exposure concentration), along with all those that displayed a mucoid phenotype, were passaged daily onto drug-free LB agar for 5 days at 37°C, with one passage performed at RT to check for the mucoid colony phenotype. Under conditions with little to no change in MIC after 3 days, such as the no-drug control, isolates that displayed no change in resistance were selected for passaging. For each isolate, a single colony was cultured overnight in drug-free media and frozen with 50% glycerol. The MIC against ceftriaxone was assayed for these mutants as before using saturated cells, along with a WT sample. An unpaired two-tailed *t* test was used for statistical analysis to compare mean fold change in MIC between exposure groups. A *P* value of <0.05 was considered statistically significant.

### Motility assays.

Swimming motility was assayed on 0.3% LB agar plates without antibiotic at 30°C, as described by Partridge and Harshey (71), using 5 μl of a saturated culture grown at 37°C. The diameter of radial movement (indicated by visible growth of cells) was measured after ∼20 h using a ruler. A ratio between motility of the WT and that of the mutant was calculated (diameter of mutant/diameter of WT). Ratio metric values provided a comparable and consistent measurement between experiments (Fig. S1C, panel ii). An unpaired two-tailed *t* test was used for statistical analysis to compare mean motility measurements between resistant and susceptible isolates. A *P* value of <0.05 was considered statistically significant.

### Whole-genome sequencing.

Whole-genome sequencing was performed on all selected 3-day-exposed isolates (described above) that were subsequently passaged on drug-free media for 5 days (39 total). DNA was extracted using the Qiagen blood and tissue extraction kit according to the manufacturer’s protocol. Library preparation and paired-end whole-genome sequencing were performed at the Microbial Genome Sequencing Center (Pittsburgh, PA) using the Illumina NextSeq 2000 platform. Data processing and analysis were performed using CLC Genomics Workbench 21.0.3 (Qiagen). Briefly, sequencing reads from unaligned FASTQ files were aligned to the reference E. coli MG1655 genome FASTQ file (NC_ 000913.3) downloaded from the NCBI using the function “Map Reads to Reference” with default settings. After mapping, detection of SNVs, insertions, and deletions was performed using the “Basic Variant Detection” tool with a >95% frequency-across-read cutoff. PROVEAN (72) was used to predict whether a missense SNV was deleterious or neutral.
