# Supplementary figures and images for: Identification of Multiple Low-Level Resistance Determinants and Coselection of Motility Impairment upon Sub-MIC Ceftriaxone Exposure in Escherichia coli
Source: mSphere. 2021 Nov 17;6(6):e00778-21. doi: 10.1128/mSphere.00778-21 (PMC8597738; doi:10.1128/mSphere.00778-21)

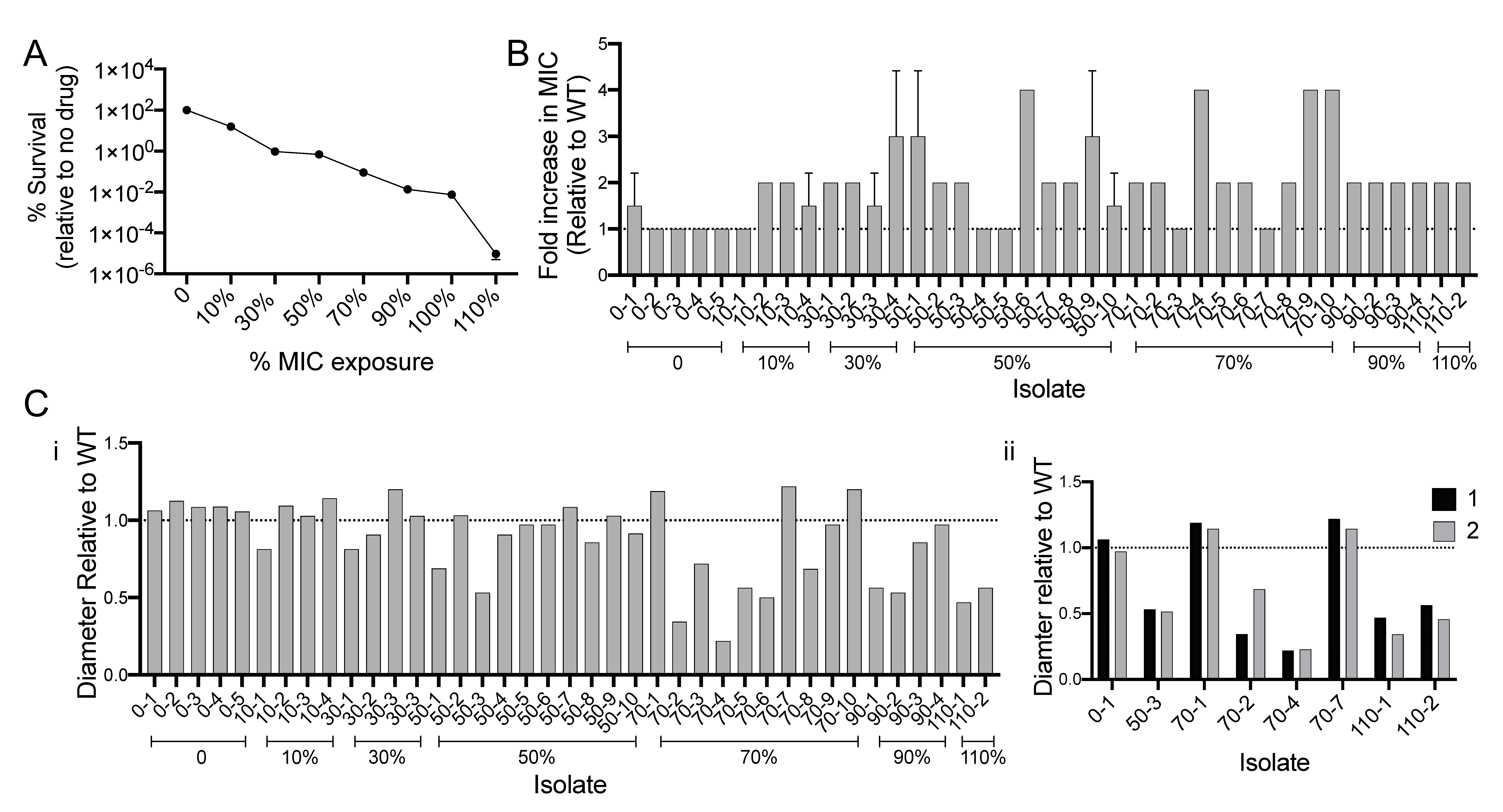

Supplement: FIG S1 [file msphere.00778-21-s0002.tif]
